# Quality Tool School: Improving the Delivery of Quality Improvement Education in a Children’s Hospital

**DOI:** 10.1097/pq9.0000000000000680

**Published:** 2023-09-28

**Authors:** James Gallup, Don Buckingham, Kevin Dolan, Charlie Macias

**Affiliations:** From the * Nationwide Children’s Hospital, Center for Clinical Excellence, Columbus, Ohio.; † Nationwide Children’s Hospital, Columbus, Ohio.

## Abstract

**Background:**

In 2013, Nationwide Children’s Hospital’s (NCH) Quality Tool School (QTS) was created as an initial Quality Improvement educational series, composed of three separate classes, totaling 5.5 hours of hands-on QI training. QTS complemented the NCH 40-hour Quality Improvement Essentials course.

**Methods:**

Over 10 years, the series went through three phases of aims: *Phase 1*: develop and implement three core courses (Project Tools, Excel, and Control Charts); *Phase 2*: have participants complete the entire series of all three classes; *Phase 3*: have participants who complete the entire series of all three classes demonstrate the application of learning through involvement in a quality improvement project.

**Results:**

Since initiation, QTS has provided an educational entry point for 1428 NCH employees to participate in QI projects and teams. QTS has shown statistically significant improvement in 2 of the 3 principal aims. The Phase 1 *metric of average monthly one-class participation completion percentage* showed a statistically significant centerline shift from 9 to 16 students in October 2018. The Phase 3 metric *Percentage of QTS participants completing the QTS series of classes and then participating in a QI team* began in 2016 with a baseline of 42%. A centerline shift from 42% to 63% occurred in Q4 2018.

**Conclusions:**

QTS can provide QI education to healthcare system employees using limited resources. Organizations that strategically integrate a culture of QI into core beliefs can realize substantial improvement gains.

## INTRODUCTION

A systematic approach to improving quality and safety metrics through disseminating quality improvement (QI) methodology and high-functioning improvement teams is a strategic goal for most institutions. QI education fosters long-term improvement capabilities and skills for the staff and the organization.^1^

Nationwide Children’s Hospital’s Center for Clinical Excellence (NCH-CCE) has adopted strategies from the Institute for Healthcare Improvement,^2^ Lean, and Six Sigma, adapting processes initially designed within industries outside healthcare to improve organizational quality metrics. Effective QI education for all levels of staff with multiple entry points is a foundational component in achieving NCH strategic goals.^3^

QI team education is currently available in many formats. In healthcare, the Institute for Healthcare Improvement has championed the spread of the Deming Cycle (Plan-Do-Study-Act) and many forms of QI education.^4^ Despite several successes, an opportunity exists to increase the number of internal leaders to champion QI projects and understand QI methodology.^5^ The key to this opportunity is to expand current QI educational offerings and benchmark against models in all industries, including healthcare,^6^ to enhance our QI systems.

NCH created Quality Tool School (QTS) (curriculum content in Table 1) in 2013. QTS is an entry-level QI course for NCH employees participating in QI projects and teams. This report includes a description of the initial development and delivery of QI education with three distinct phases of goals, a discussion of the potential to expand to other clinical settings, and a description of how this approach can complement existing QI educational activities in a healthcare delivery system.

**Table 1.** **Description of Classes in the Session (Three Total Classes**)

QTS Project Tools (Class 1)
**Two-hour Class:** This is the express class that covers current situation analysis, brainstorming quality tools, and key driver development. If you have a project that needs done now, this class will help you get to a key driver diagram quickly.
**Quality Tool School Excel Basic Skills (Class 2a**)*
**One-hour Class:** This is a 1-hour, hands-on introductory class on using Excel for data analysis. Class content includes Excel basics, introduction to mathematical calculations in Excel, and design of a check sheet for a short cycle PDSAs.
**Quality Tool School Excel Intermediate Skills (Class 2b**)*
**One-hour Class:** This is a 1-hour, hands-on intermediate class on using Excel for data analysis. Class content includes intermediate skills (sorting, filtering, creating pivot tables, creating charts and using advanced functions) needed to analyze QI data.
**Quality Tool School Control Charts —1.5 hours (Class 3**)
**One-and-a-half-hour Class:** This is a 1.5-hour, hands-on introductory class to using NCH Pareto templates, NCH control chart templates, and basic control chart theory.

* From 2013 to 2019, Excel was taught as a 2-hour class. Starting in 2019, it was taught as 2, 1-hour classes (basic and intermediate), and experienced Excel users could opt out of the basic level.

At its inception, NCH’s initial QTS aim was to increase the number of employees starting the QI learning process by completing at least one class. We added a second aim tracking participant completion of the entire course, and later a third aim assessing employees who completed the course as part of participation in a QI team. QTS was designed to complement the Quality Improvement Essentials (QIE) class that targeted improvement leaders in the organization.

NCH previously published a study evaluating the 40-hour NCH QIE course 2017^5^; That study details how QIE dramatically improved participant QI competency as (measured by pre- and post-participant assessments) and improved individual QI productivity. QIE was highlighted by:

- In-class multi-disciplinary learning groups;
- The required final presentation of QI projects;
- Creation of QI project teams;
- 6-month follow-up on QI projects to ensure retention of QI skills by training participants.

NCH QIE was one of the first QI leadership programs in health care. Combining QIE and QTS was the beginning of a comprehensive QI educational model. Another comprehensive healthcare-specific model for organizations to consider is the QI training model developed at Cincinnati Children’s Hospital.^6^

## METHODS

### Context

Located within Columbus, Ohio, Nationwide Children’s Hospital is a quarternary pediatric healthcare facility and is one of the largest pediatric hospitals in the United States. In 2013, at project initiation and implementation of QTS, the hospital employed approximately 5000 employees. QTS was intended to complement a larger course available at our institution, QIE, as an initial educational step. QIE is taught by hospital QI leadership, targeting primarily hospital leaders (physicians, nurses, and administrators). This course includes approximately 40 hours of scheduled class time and requires that participants complete a QI project throughout the course with an assigned QI coach. In contrast, QTS has an open format and provides approximately 5.5 hours of hands-on QI training. QTS is available to any employee and does not have prerequisite requirements.

### Interventions

#### Phase 1 (2013 to present)

In 2013, an NCH QI team member developed and started teaching QTS Project Tools (Table 1). We created additional courses for control-chart-based content and Microsoft Excel in 2014 (Table 1). Initial goals were to consistently fill classes monthly (Fig. 2, Measure A), to make classes highly interactive, and to make the course available to any interested employee. We offered 5.5 hours of continuing education (CE) credits to increase nursing participation. Over the next 7 years, the expansion of class size from an average of 9 to 16 students was fostered by speaking with internal hospital groups and leaders. Attendees were not required to complete all of the learning modules, allowing flexibility to our target attendees.

**Fig. 1.**
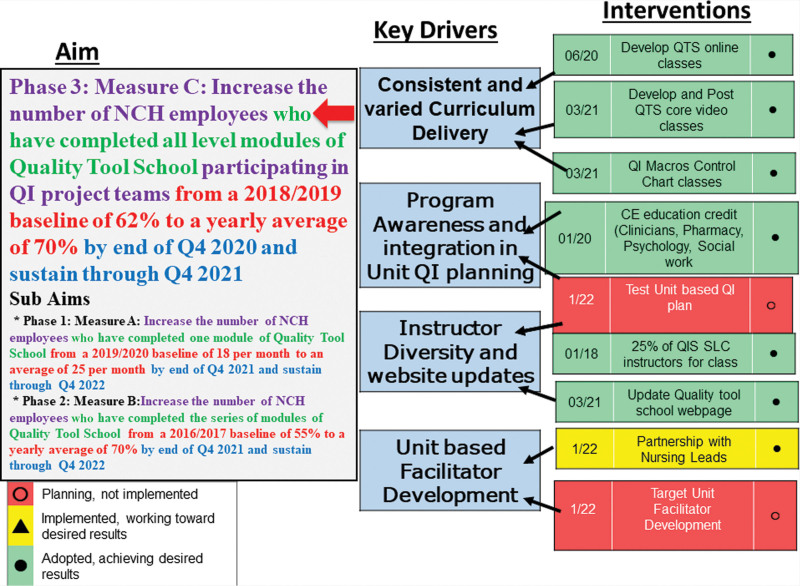
Key driver diagram.

#### Phase 2 (2016 to Present)

Based on our first formal report and discussions about improving QI project development with the chief nursing and chief medical officers, an additional focus was to have participants finish the entire series of learning modules in preparation for leading QI projects (Fig. 3, Measure B). We improved ease of class registration by collaborating with the electronic learning management system to post enrollment online. We expanded the faculty to include additional QI methodology experts to add variety to the presentations. Based on instructor feedback, the team modified the format to use a standard 7-step module starting in November 2017. Also in late 2017, the team modified the control chart class based on prior participant feedback. Further, the Behavioral Health service line started integrating QTS into yearly QI education. In late 2018, we started offering education credit for physicians who participated.

**Fig. 2.**
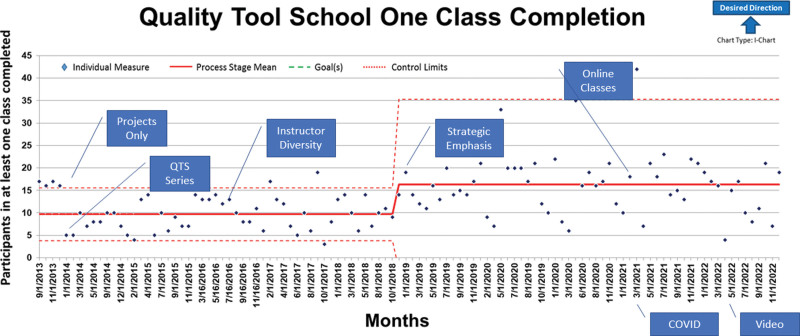
Phase 1 Control Chart (Measure A): (7)

**Fig. 3.**
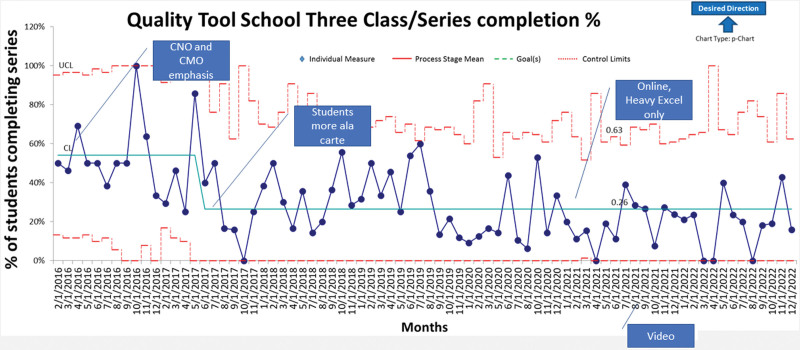
Phase 2 Control Chart (Measure B): (7)

#### Phase 3 (2019 to Present)

**Fig. 4.**
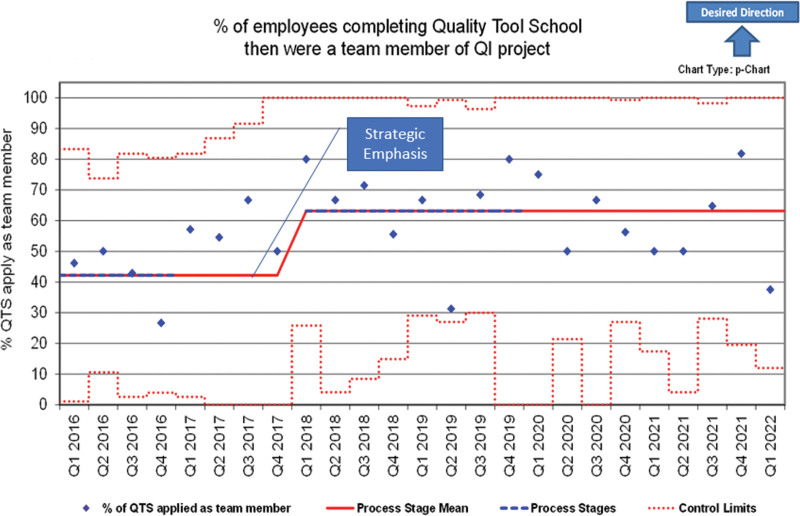
Phase 3 Control Chart (Measure C): (7)

To meet CE educational credit requirements, we added a focus area (Fig. 4) measuring QI team participation for employees who completed the entire QTS series of courses. Also, we built a formal Key Driver diagram (Fig. 1) to develop a strategy around the three targeted aims. To help expand the number of students applying the QTS tools to QI teams, we increased the instructor team from 4 to 10. In May 2020, in response to in-person limitations imposed by the COVID-19 pandemic, we adapted to a virtual learning model with classes taught online by an instructor. Based on learning from these online sessions, we developed a self-paced video learning series and implemented it in September 2020.

### Measures

#### Phase 1

In 2013, initial measures included the number of employees completing at least one class and the percentage of participants rating the class as excellent on the class evaluation (Table 2: Phase 1). The first goal was to have at least 10 different participants per month take a least 1 of the 3 classes in the series (Table 2: Phase 1). We tracked one-class completion (Phase 1) using a control chart (Fig. 2, measure A). The second goal of at least 90% of participants rating the class as excellent was a requirement for nursing CE credit.

**Table 2.** Class Measures

Phase	Goal	9/2013 to 12/2015	1/2016 to 12/2017	1/2018 to 3/2022
Phase 1 (Measure A)	Average number of participants per month completing at least one QTS class and rating the class excellent (one class completion %)	9	11	16
Phase 2 (Measure B)	Average monthly % participants completing the Three classes/Quality Tool School Series (Project Tools, Excel, Control Chart)	Not measured	45%	25%
Phase 3 (Measure C)	% of employees completing the QTS Series (Phase 2 goal) that were a team member of a QI project team (Phase 3)	Not measured	48%	60%

#### Phase 2

Based on feedback in 2016 from hospital leadership, the team added the goal of increasing the percentage of employees completing the recommended sequence of three classes: Project Tools, Microsoft Excel, and Control Charts (Table 2: Phase 2) and monitored progress on a control chart (Fig. 3, Measure B). Every month, the team compared the percentage of participants completing the three-class series to the rate of participants completing only 1 class.

#### Phase 3

A final measure, the percentage of participants completing the entire sequence of QTS classes who then became a member of a QI improvement team (Table 2: Phase 3), was added in 2018 to assess the application of QI skills as requested by the CE team. A control chart displayed progress (Fig. 4, Measure C).

### Analysis

The team collected data on class attendance and satisfaction. We used that data to create control charts and to track overall averages for all measures except measure C. A yearly survey was sent to participants and QI consultants to confirm a team member’s application of QI tools in a QI project. We used this survey data for measure C.

The team used statistical process control to evaluate whether changes implemented via interventions were meaningful.^7^ We loaded data into proprietary control charting templates,^8^ which allowed the team to study process changes over time. The team evaluated each outcome monthly, except for measure C, which was assessed yearly. We used American Society for Quality criteria^7^ for adjusting control chart centerlines and control limits. The team adjusted interventions based on statistical process control data and feedback received during Plan-Do-Study-Act cycles. We adjusted interventions as needed. Every year, the team submitted annual reporting to the QTS steering committee and senior quality leadership.

### Ethical Considerations

The institutional review board determined that this project was QI and not human subject research. Therefore, it did not require institutional review board review and approval per institutional policy. The Standards for Quality Improvement Reporting Excellence (SQUIRE) 2.0 guidelines were used to prepare this article.^9^

## RESULTS

### Phase 1

From 9/2013 to 9/2018, QTS had an average of 9 new participants per month, as represented by the centerline average on Measure A (Fig. 2). A centerline shift from 9 to 16 occurred in October 2018 (*P* = 0.000 based on a 2-sample *t* test). In 2021 and 2022, QTS averaged over 200 one-class participants per year. May 2020 is an outlier with 33 new participants, coinciding with the start of online classes (Fig. 2). At the end of 2019, 33% of participants were nurses (n = 43), and nearly 60% of the participants had frontline positions (nursing, administrative support, laboratory, respiratory therapy, and medical support; n = 99). Since 2013, we have trained over 1428 students internally, meeting the need of nearly 11000 NCH Employees, as of 2022.

### Phase 2

Starting in 2016, three-class series completion was measured, with an initial 2016 centerline of 54% of participants completing the entire sequence of classes (Fig. 3). Participation decreased starting in the middle of 2017 to an average of 26% of participants completing the series. The centerline has maintained an average of 26% from February 2018 to the present.

### Phase 3

In 2016, we started the Phase 3 measure of QTS participants completing the QTS series of classes and then participating in a QI team (Fig. 4). A centerline shift from the 2016 baseline of 42% to 63% occurred in Q4 2018 (*P* = 0.001 using a 2-sample *t* test).

## DISCUSSION

NCH’s QTS provides a meaningful educational experience to help introduce participants to QI science. We supported QTS only with part-time resources, and we have no dedicated project support structure or required project update for participants. We based QTS training modules on the Mager instructional design.^10^ This includes demonstration, practice, and feedback within the class structure for fundamental quality tools needed to support QI projects.^4^ We designed all live QTS classes to obtain Level 1 and 2 training effectiveness criteria (Phillips).^11,12^ These criteria measure positive reaction, followed by learning and confidence through actual practice of the QI skill in class (eg, developing an aim statement). Measurement of application of training (Level 3 Phillips) was limited to measuring team participation for employees completing a series of three classes (Fig. 4). To measure application, impact, and return on investment, additional resources would have been required. These include project submittal/approval for strategic integration, assigned coaches, and required project presentations. These resources were part of NCH’s QIE,^5^ which allowed for a deeper measure of effectiveness in designing QI education.

In some organizations, introductory programs like QTS are part of a comprehensive improvement education system design that is executive, managerial, and frontline focused (integrated design). The goals of those laddered educational systems are associate empowerment; thus, resources needed to achieve deeper learning (Phillips)^11,12^ are dedicated. Examples of institutions with comprehensive QI education systems include the Mayo Clinic, Franciscan St. Francis Hospital, Honda of America, and Toyota of American Manufacturing.^13–19^

We designed QTS to achieve three goals. The first goal was as an entry point to QI education for any employee. The results achieved in measure A (Fig. 1) show that NCH has progressed and that one-class participation has good leadership support. The contributing factor involved in the centerline shift was an increased strategic emphasis by hospital departments (eg, Behavioral Health, Primary Care, and Pharmacy). The numbers for one-class participation have continued to be strong following the introduction of an online Excel class, which was added due to the COVID-19 pandemic. The second goal of series completion (Fig. 2, Measure B) has not shown targeted improvement. Discussions to integrate more into employee development and yearly unit quality planning has not been adopted, which may have impacted results. The final goal of application of QTS tools in an improvement team (Figure 2, Measure B) shifted initially and held steady. This was due to the strategic emphasis by departments on adopting the original design of completing the QTS series and applying tools with an improvement team.

## CONCLUSIONS

QTS is an effective means of providing initial QI education to healthcare system employees at all levels in the organization. Using limited resources, other organizations can develop this fundamental piece of an education system. If organizations strategically integrate a culture of QI into core beliefs, substantial improvement gains are possible.

## ACKNOWLEDGMENTS

The authors would like to recognize and thank past and present members of the Center for Clinical Excellence who have served as Quality Tool School instructors and leaders of the Center for Clinical Excellence for their support of the program.

## DISCLOSURE

The authors have no financial interest to declare in relation to the content of this article.
